# Changing Antimicrobial Resistance Trends in Kathmandu, Nepal: A 23-Year Retrospective Analysis of Bacteraemia

**DOI:** 10.3389/fmed.2018.00262

**Published:** 2018-09-19

**Authors:** Raphaël M. Zellweger, Buddha Basnyat, Poojan Shrestha, Krishna G. Prajapati, Sabina Dongol, Paban K. Sharma, Samir Koirala, Thomas C. Darton, Christine Boinett, Corinne N. Thompson, Guy E. Thwaites, Stephen Baker, Abhilasha Karkey

**Affiliations:** ^1^Wellcome Trust Major Overseas Programme, Oxford University Clinical Research Unit, The Hospital for Tropical Diseases, Ho Chi Minh City, Vietnam; ^2^Oxford University Clinical Research Unit, Patan Academy of Health Sciences, Kathmandu, Nepal; ^3^Centre for Tropical Medicine and Global Health, Oxford University, Oxford, United Kingdom; ^4^Global Antibiotic Resistance Partnership, Centre for Disease Dynamics Economics and Policy, Washington, DC, United States; ^5^Patan Academy of Health Sciences, Patan Hospital, Kathmandu, Nepal; ^6^Sheffield teaching hospitals NHS trust foundation and the University of Sheffield, Sheffield, United Kingdom; ^7^The Department of Medicine, University of Cambridge, Cambridge, United Kingdom

**Keywords:** bloodstream infections, bacteraemia, blood culture, surveillance, community acquired, antimicrobial resistance, enterobacteriaceae, Nepal

## Abstract

A comprehensive longitudinal understanding of the changing epidemiology of the agents causing bacteraemia and their AMR profiles in key locations is crucial for assessing the progression and magnitude of the global AMR crisis. We performed a retrospective analysis of routine microbiological data from April 1992 to December 2014, studying the time trends of non-*Salmonella* associated bacteraemia at a single Kathmandu healthcare facility. The distribution of aetiological agents, their antimicrobial susceptibility profiles, and the hospital ward of isolation were assessed. Two hundred twenty-four thousand seven hundred forty-one blood cultures were performed over the study period, of which, 30,353 (13.5%) exhibited growth for non-contaminant bacteria. We observed a significant increasing trend in the proportion of MDR non-*Salmonella* Enterobacteriaceae (*p* < 0.001), other Gram-negative organisms (*p* = 0.006), and Gram-positive organisms (*p* = 0.006) over time. Additionally, there was an annual increasing trend in the proportion of MDR organisms in bacteria-positive blood cultures originating from patients attending the emergency ward (*p* = 0.006) and the outpatient department (*p* = 0.006). This unique dataset demonstrates that community acquired non-*Salmonella* bacteraemia has become an increasingly important cause of hospital admission in Kathmandu. An increasing burden of bacteraemia associated with MDR organisms in the community underscores the need for preventing the circulation of MDR bacteria within the local population.

## Introduction

Bacteraemia continues to be a common cause of febrile illness worldwide, and is associated with substantial morbidity and mortality (1, 2). The spectrum of organisms causing bacteraemia is variable and highly setting-specific (2–4). The disease severity and outcome of bacteraemia can also vary significantly, and are related to multiple factors, including the genetic composition of the infecting bacteria, susceptibility of the host, and the clinical management of the patient (5–7).

In recent years, there have been substantial changes in the aetiological agents of bacteraemia and their associated antimicrobial susceptibility profiles (5, 6, 8). The rapid administration of an effective antimicrobial is imperative for the clinical management of bacteraemia; however increasing antimicrobial resistance (AMR) restricts treatment options. The sustained usage of broad-spectrum antimicrobials has become associated with the emergence, selection, and circulation of invasive bacterial pathogens exhibiting AMR to many empirically prescribed antimicrobials (9). Therefore, a comprehensive longitudinal understanding of the changing epidemiology of the agents causing bacteraemia and their AMR profiles in specific locations is crucial for assessing the scale of the global AMR crisis, revising rational management strategies, and improving treatment guidelines (10, 11).

In Nepal, febrile disease is a common reason for seeking medical attention (12). Like in many other low-income countries, much of the febrile disease burden in Nepal is associated with community-acquired infections. Invasive *Salmonella* serovars Typhi and Paratyphi A, now with resistance against fluoroquinolones, remain the organisms most frequently isolated from the blood of febrile patients in the Kathmandu Valley (12–14). However, invasive *Salmonella* are less adept at acquiring resistance to multiple antimicrobials than other indicator organisms and may not reflect general trends in AMR (15). Furthermore, *Salmonella* Typhi and Paratyphi A are well established and well described pathogens in the Kathmandu Valley, but little has been reported for non-*Salmonella* associated bacteraemia. To investigate the epidemiology and AMR profile of agents causing bacteraemia in this setting, we performed a retrospective analysis on 23 years of data regarding the etiology of non-*Salmonella* associated bacteraemia at Patan hospital, a major general hospital in the Kathmandu Valley.

## Methods

### Ethics statement

This study used anonymised data, originating from the microbiology laboratory of Patan Hospital in Kathmandu, consisting of culture result, isolated organism, ward in which the blood sample was taken, and associated AMR profile. This study was, therefore, a component of the routine surveillance measures for infection control at Patan Hospital and local ethical approval and individual written informed consent were not required. However, a written permission, for access and analysis of the data, was sought and obtained from the Hospital.

### Study design

This study was a retrospective analysis of routine microbiology laboratory results of all blood cultures taken at Patan Hospital between April 1992 and December 2014. It is one of three general hospitals in the greater metropolitan area of Kathmandu. The hospital had a capacity of 138 beds in 1992, 350 beds in 2014, a current capacity of 592 beds, and provides emergency and elective services to outpatients (~200,000 outpatient visits per year) and inpatients, 90% of which live in Lalitpur Sub Metropolitan City LSMC.

Systematic criteria concerning which patients had a blood culture performed were not used during the course of the study. However, a blood culture was generally performed on patients in whom a bacterial infection was suspected on the basis of a fever (>38°C) or evidence of sepsis on the basis of the presence of two or more of the following criteria: fever (>38°C) or sub-normal temperature (<36°C), tachycardia, tachypnoea, elevated white cell count (>12,000 cells/mm^3^), or depressed white cell count (<4,000 cells/mm^3^). Review of the microbiological standard operating procedures over the analysis time period suggest no substantive changes in the application of these criteria. Bacteraemia was defined as isolation of at least one clinically relevant pathogen from one blood culture drawn from a patient with an indicative clinical syndrome. Organisms of the same species with the same antimicrobial susceptibility profile isolated from the same patient (matching hospital ID number) were removed from analysis.

### Microbiology procedures and antimicrobial susceptibility testing

The majority of blood cultures were performed manually by inoculating 3–5 mL (pediatric patients) or 5–8 mL (adult patients) of blood into 30–50 mL of media containing tryptone soya broth and 0.05% sodium polyanetholesulfonate. An automated BACTEC (Becton Dickinson, MD, USA) system was introduced for cultures on blood taken from those aged <14 years from 2005 onwards. Blood samples from children were inoculated into BACTEC Peds plus bottles following the manufacturers recommendations (Becton Dickinson, MD, USA). All inoculated blood bottles were incubated at 37°C and examined daily over 7 days for bacterial growth, or until flagged in the automated system. For all conventional bottles, irrespective of whether turbidity developed or not, the inoculated broth was sub-cultured and Gram stained on sixth day of incubation. Organisms isolated from the primary cultures were sub-cultured onto sheep blood agar and chocolate agar. Plates were incubated at 37°C for 48 h. Organisms were identified by standard methods including API20E identification kits (Bio-Mérieux, Craponne, France) and specific antisera where possible. Coagulase negative staphylococci were reported only for neonates as pathogens and as contaminants for the rest of the population. Gram-negative rods that were not identifiable through biochemical tests were reported as Gram-negative rods.

Antimicrobial susceptibility testing was performed at time of isolation by the modified Kirby-Bauer disc diffusion method. Zone size interpretations were initially performed following the guidelines provided in the antimicrobial disc packaging; from 2003 onwards, susceptibilities were inferred by following the appropriate CLSI guidelines. A range of antimicrobials were tested, this was largely dependent on the period of isolation (Table S1).

### Statistical analysis

For analysis purposes, resistant and intermediate organisms were grouped as “non-susceptible.” Multi-drug resistance (MDR) was defined as “acquired non-susceptibility to at least one agent in three or more antimicrobial categories” (16). The categories used in this study are described in Table S2. As current practice, laboratory results reporting resistance to an antimicrobial for which an organism was intrinsically resistant were not taken into account when generating the MDR profile. Intrinsic resistance were determined according to the 2014 Clinical and Laboratory Standards Institute (CLSI) guidelines (Table S3) (17). Increasing or decreasing trends in isolated organisms and AMR profiles were measured using Cox and Stuart test for trends (R-package “randtrends”). Davies' test (R-package “segmented”) was used to detect a non-constant regression parameter (i.e., change in slope) when the percentage of MDR was regressed on years. All analyses were performed using the statistical software R version 3.3.2 (18).

## Results

### General observations

From April 1992 to December 2014, a total of 224,741 blood cultures were performed at Patan Hospital, of which 173,892 (77.4%) were culture negative, and a further 20,410 (9.1%) contained a contaminant organism. A non-contaminant fungal pathogen was identified in 86 blood samples, the remaining 30,353 (13.5%) blood cultures exhibited growth for non-contaminant bacteria (Table 1). The most frequently isolated organisms were *Salmonella* Typhi and *Salmonella* Paratyphi A, associated with 44.8% (13,592/30,353) and 20.0% (6,057/30,353) of all bacteria-positive blood cultures, respectively (Table S4).

**Table 1:** Summary of the blood cultures performed between April 1992 and December 2014.

**Blood cultures (*n* = 224,741)**	**N**	**Proportion of cultures (%)**
Culture negative	173,892	77.37
Contamination^*^	20,410	9.08
Fungi	86	0.04
Culture bacteriologically positive	30,353	13.51
*Salmonella enterica*	19,857	8.84
Enterobacteriaceae *non-Salmonella*	4,029	1.79
Gram-negative (other)	2,087	0.93
Gram-positive	4,380	1.95

* *Organisms that were classified as contaminants included Bacillus spp., Micrococcus spp., Coagulase negative staphylococci excluding those from neonates and diptheroids*.

The most frequently isolated non-*Salmonella* organisms were (in decreasing frequency) *Enterobacter* spp., *Acinetobacter* spp., Coagulase negative *Staphylococci, Escherichia coli, Klebsiella* spp. (including both *K. pneumoniae* and *K. oxytoca*), *Staphylococcus aureus*, Non-haemolytic *Streptococci*, and *Streptococcus pneumoniae* (Table S4). Between 1992 and 2014, the total number of blood cultures performed increased, but the proportion of blood samples exhibiting bacterial growth showed a decreasing trend (*p* < 0.001; Cox and Stuart test) (Figure S1).

### Time trends of gram-positive and gram-negative bacteria

**Figure 1:**
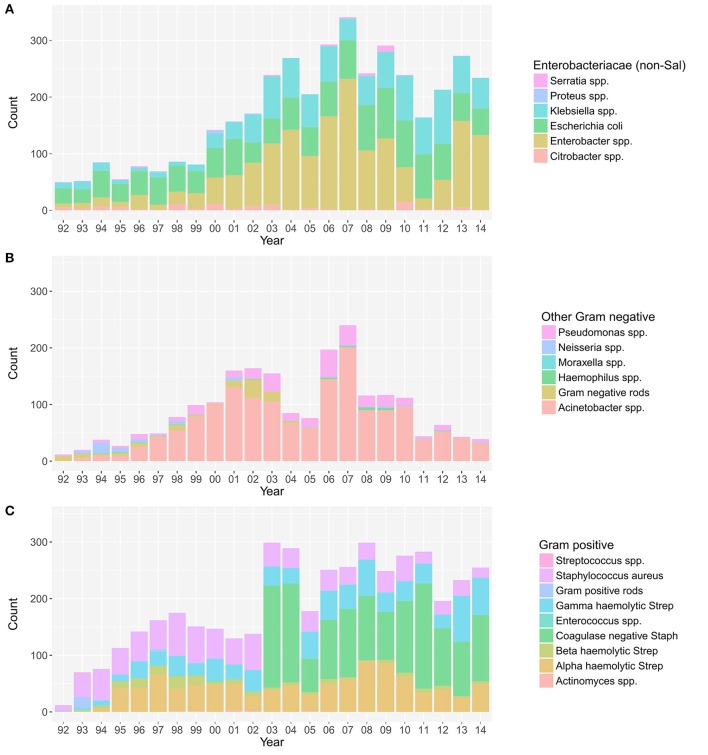
The annual distribution of the aetiological agents associated with bacteraemia. The distribution of non-*Salmonella* Enterobacteriaceae **(A)**, other Gram-negative organisms **(B)**, and Gram-positive organisms **(C)** isolated from positive blood cultures in this single healthcare facility between April 1992 and December 2014.

The annual distribution of the aetiological agents associated with bacteraemia, stratified by non-*Salmonella* Enterobacteriaceae, other Gram-negative organisms, and Gram-positive organisms (19), is shown in Figure 1. The absolute annual counts of Enterobacteriaceae and Gram-positive organisms showed an increasing trend from 1992 to 2014 (*p* < 0.001, Cox and Stuart test). The proportion of non-*Salmonella* Enterobacteriaceae isolated from 1992 to 2014 additionally exhibited an increasing annual trend (*p* < 0.001, Cox and Stuart test) (Figure S2). No such trend was observed for other Gram-negative organisms, which exhibited a peak in 2007.

### Time trends of multi-drug resistant organisms

Over the study period we identified a significant increasing trend in the proportion of MDR non-*Salmonella* Enterobacteriaceae (*p* < 0.001; Cox and Stuart test), other Gram-negative organisms (*p* = 0.006), and Gram-positive organisms (*p* = 0.006) isolated (**Figure 3** and Figure S3). The proportion of MDR organisms was regressed on time to assess the annual increase in the proportion of MDR organisms. We additionally assessed the presence of breakpoints in the regression to identify any specific years that were associated with a major change in the rate of the increase in MDR percentage. For the non-*Salmonella* Enterobacteriaceae and other Gram-negative organisms, there was no breakpoint in the regression plots, signifying a constant increase in the proportion of MDR organisms of 1.5 and 1.9% per year, respectively (**Figure 3**). Conversely, the Gram-positive organisms exhibited a breakpoint in 2010/2011, indicating acceleration in the annual increase of the proportion of MDR organisms from 2011 onwards (1992–2010: 1.26% annual increase in the proportion of MDR organisms, 2011–2014: 7.61% annual increase).

**Figure 2:**
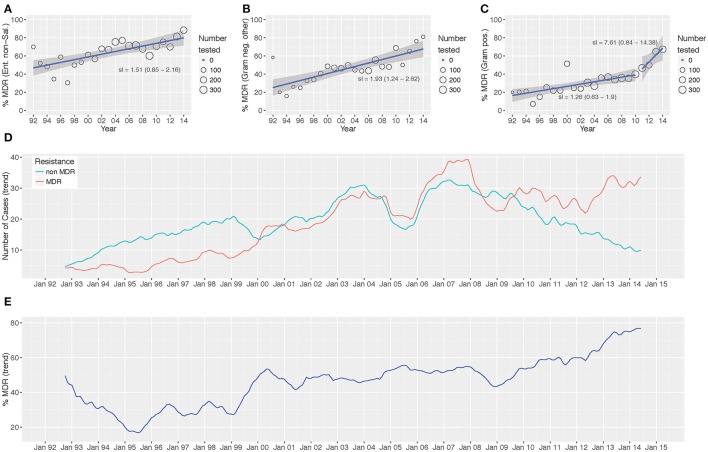
Antimicrobial resistance trends from 1992 to 2014. The proportion of MDR Enterobacteriaceae non-*Salmonella* **(A)**, other Gram-negative organisms **(B)**, and Gram-positive organisms **(C)** isolated between 1992 and 2014. Time trends of MDR vs. non-MDR isolates [**(D)** count and **(E)** distribution]. Linear trend lines (blue) and 95% CI for the regression lines (shaded) are overlaid; the slopes of the regression line (with 95% CI) are indicated **(A-C)** two regression lines are present in **(C)** to highlight the “breakpoint” in the slope.

On investigating the time trends for MDR and non-MDR organisms we identified three distinct periods during which: (i) the number of isolated non-MDR organisms was greater than the number of MDR organisms (1992–2000, MDR/non-MDR <1), (ii) the number of isolated non-MDR organisms and MDR organisms was approximately equal (2000–2010, MDR/non-MDR≈1), and (iii) the number of MDR organisms was greater than the number of non-MDR organisms (2010–2014, MDR/non-MDR > 1) (Figure 2).

### The origins of multi-drug resistant organisms

**Figure 3:**
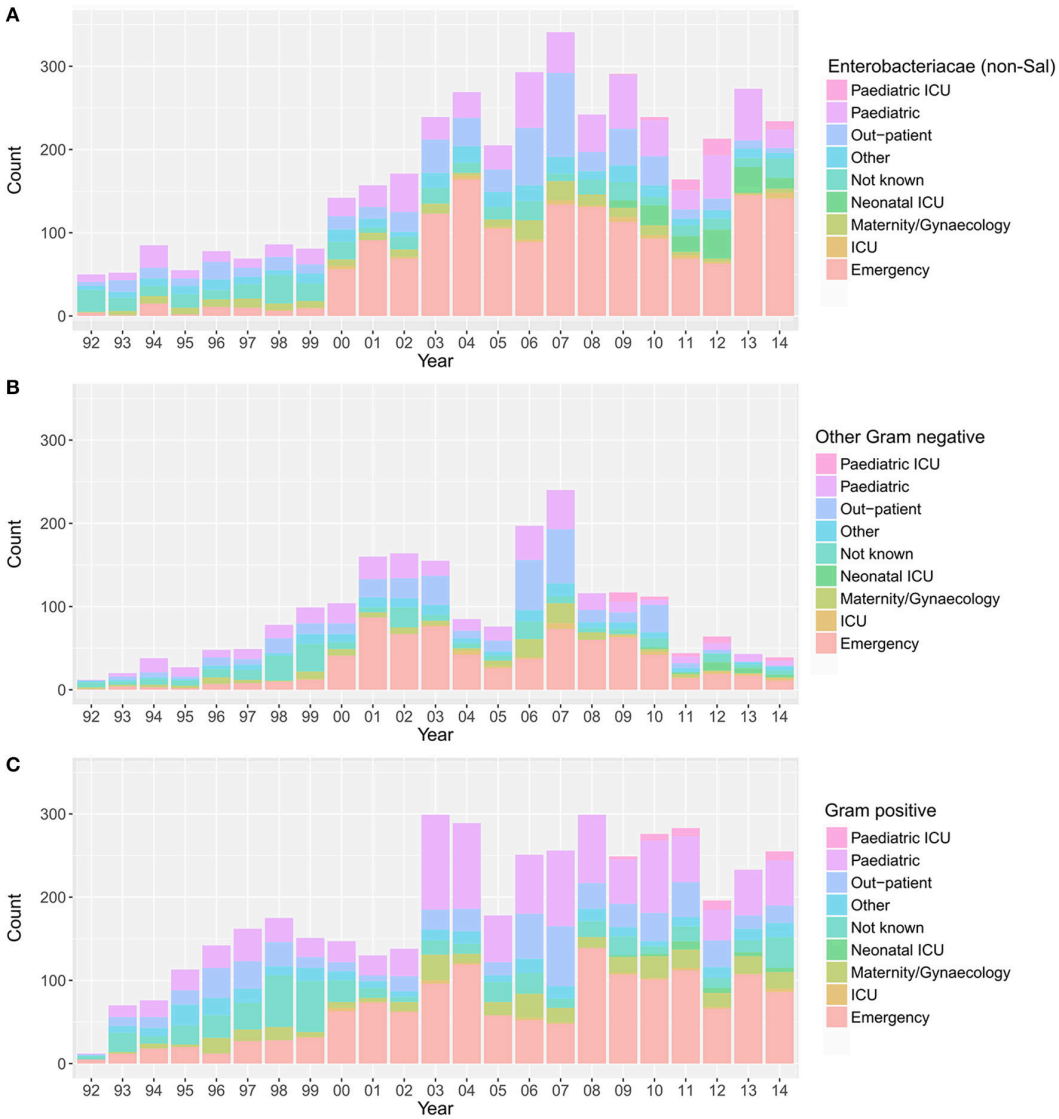
The annual distribution of wards in which the positive blood samples originated. Plots showing the hospital wards from which the positive blood samples originated between April 1992 and December 2014; Enterobacteriaceae non-*Salmonella* **(A)**, other Gram-negative organisms **(B)**, and Gram-positive organisms **(C)**. Maternity, birthing, post-partum and gynecology wards were grouped under “maternity/gynecology,” pediatric and nursery wards were grouped under “pediatric,” and orthopedic, private, medical, and surgical wards were grouped under “other”.

Over the investigative period, the majority of positive blood cultures originated from patients attending the emergency department (3,808/10,496; 36.3%), the pediatric ward (2,217/10,496; 21.1%), and the outpatient department (1,528/10,496; 14.6%). The yearly distribution of wards from where the various groups of organisms were isolated is shown in Figure 3. The proportion of blood cultures positive with Enterobacteriaceae from patients attending the emergency department (in comparison to other wards in the hospital) showed an increasing trend between 1992 and 2014 (*p* = 0.03; Cox and Stuart test).

Finally, we assessed the relative distribution of MDR and non-MDR organisms in different hospital wards (Figure S4). Over the entire period, for both the MDR and the non-MDR organisms, the majority of organisms were isolated from patients attending the emergency department (MDR: 36.4%; 1,938/5,319, non-MDR: 36.3%; 1,859/5,127), followed by the pediatric ward (MDR: 22.7%; 1,206/5,319, non-MDR: 19.6%; 1,004/5,127), and the out-patient department (MDR: 12.5%; 663/5,319, non-MDR: 16.7%; 857/5,127). The number of bacteriologically positive cultures originating from the emergency department increased throughout the study period. There was an increasing trend in the proportion of MDR organisms in all bacteria-positive cultures in the emergency ward (*p* = 0.006, Cox and Stuart test), the pediatric department (*p* = 0.006, Cox and Stuart test), and the outpatient department (*p* = 0.006, Cox and Stuart test).

## Discussion

In this retrospective analysis of bacteraemia from a single hospital in Kathmandu, we report a 13.5% bacterial isolation rate from blood cultures, which is comparable with other bacteraemia studies from Nepal and elsewhere (5, 20–22). Similarly, the array of isolated organisms was similar to those reported in other studies investigating the etiology of bloodstream infections (2, 3, 22–25), and childhood septicaemia (5, 11, 25, 26). The most common Gram-negative, non-*Salmonella* organisms to be isolated from blood cultures within this population were *Enterobacter* spp., followed by *Acinetobacter* spp., *E. coli* and *Klebsiella* spp., a distribution that has been observed previously (3, 5, 23, 27). The dominance of *Enterobacter* spp. in this setting was unexpected, but there have been recent observations regarding the increasing presence of *Enterobacter* spp. bacteraemia (28). This escalation is cause for concern, as this bacterial genus presents particular challenges for the selection of optimal antimicrobial therapy due to the presence of chromosomally encoded AmpC beta-lactamases (25). Among the Gram-positive organisms (excluding Coagulase negative *Staphylococci*, which were reported for neonates only), *S. aureus*, Non-haemolytic *Streptococci* and *S. pneumoniae* were the most commonly isolated pathogens from blood cultures; this is also a common array of organisms associated with bacteraemia (5, 8, 24, 29). None of these bacteria were covered by any vaccine schedule in the country during the period of analysis.

Over the study period, there was a notable increase in bacteraemia cases originating from the emergency department, and a corresponding increase in the proportion of MDR organisms originating from patients in the emergency and outpatient departments, both of which receive patients directly from the community. This observation suggests that community-acquired bacteraemia caused by MDR pathogens is increasing and becoming a major cause of hospital admissions. These data also demonstrated a hospital-wide increase in the proportion of MDR Enterobacteriaceae, other Gram-negative organisms, and Gram-positive organisms isolated over the analysis period. In fact, from 2010, MDR isolates represented more than half of the non-*Salmonella* bacteria isolated from blood in this location. These data detail a worrying increase in MDR *Enterobacter* spp., *Klebsiella* spp., and *E. coli*, circulating both within the community and the hospital with the capacity to cause bloodstream infections. The increasing significance of *Klebsiella pneumoniae*, an organism that has already caused major outbreaks in this hospital, raises specific concerns given its ability to be a reservoir of AMR genes that can be transferred to other Enterobacteriaceae (30).

The precise reason(s) for the increase in the proportion of MDR organisms being isolated from patients in the community is unclear; we speculate that this increase is associated with an increasing population and ease of access to antimicrobials. Nepal is a landlocked country located between India and China and Kathmandu in the central valley is the capital city. Therefore, this location naturally acts as major nucleus within South Asia, with an elevated likelihood of antimicrobial resistant organisms being imported from neighboring countries. Furthermore, immigration into the capital city has increased dramatically over the last 25 years, both as a consequence of the civil war (1996–2006) and economic migration from rural areas. This increase in population has placed substantial strains on the healthcare system and the city infrastructure, making pathogen transmission more likely. Access to antimicrobials in the community without prescription and the increasing empirical use of latter generation broad spectrum antimicrobials in healthcare facilities has potentially created an increasing problem. This scenario has likely made Kathmandu a hub for the circulation of MDR organisms in the community and healthcare facilities.

The strength of this analysis is the aggregation of 23 years of data from the same healthcare facility. These longitudinal data provide a rare opportunity to observe and document changes in the epidemiology and the AMR trends of organisms associated with bacteraemia. With the exception of a recent 9-year study of bloodstream infections in Bangladesh (21), we are unaware of similar longitudinal data from other locations in South Asia with such large numbers of blood cultures and MDR time trends. However, some limitations need to be considered. Over the prolonged period, the consistency of microbiological testing (blood culture and antimicrobial susceptibility testing) needs to be ensured to allow for a meaningful comparison of trends. It is difficult, however, to wholly eliminate any changes in microbiological practices or hospital organization over time. For example, the sudden increase in the number of Coagulase negative *Staphylococci* recorded from 2003 was associated with the opening of the neonatal intensive care unit. A further limitation was the absence of demographic and clinical information in the data, thus preventing our ability to associate a specific group of organisms or change in AMR profile with patient demographics and/or clinical outcome. Similarly, treatment data and information regarding antimicrobial use prior to blood culture were not available. Lastly, as this analysis was conducted using routine hospital data, these data may not precisely reflect the epidemiology of bacteraemia in the community. However, it is important to note that the majority of positive blood cultures originated from individuals attending hospital directly from the community. Similarly, the results of this study are indicative of the situation in the Kathmandu Valley and may not reflect the situation in the rest of South Asia.

Our work illustrates worrying changes in epidemiology and AMR of bacteraemia within a healthcare facility in Kathmandu and highlights the increasing significance of non-*Salmonella* Enterobacteriaceae and Gram-positive bacteria over the last two and a half decades. This study documents the rise of AMR and MDR bloodstream pathogens in Nepal, demonstrating that MDR is not only a concern for hospital-acquired infections, but is rapidly becoming a significant problem for organisms circulating in the community. Our study will serve as a unique reference for improving clinical care, establishing treatment guidelines, and gaining a better understanding of the changing epidemiology of AMR pathogens.

## Author contributions

BB, PoS, SD, CT, SB, and AK original study design. PoS, KP, SD, PKS, SK, and AK collected the data for the study. BB, TD, GT, and AK provided data interpretation. RZ, PoS, CB, CT, SB, and AK performed the analysis. RZ, SB, and AK drafted the manuscript. RZ, BB, PoS, KP, SD, PKS, SK, TD, CB, CT, GT, SB, and AK approved the final version of the manuscript.

### Conflict of interest statement

The authors declare that the research was conducted in the absence of any commercial or financial relationships that could be construed as a potential conflict of interest.
