# Supplementary material for: Changing Antimicrobial Resistance Trends in Kathmandu, Nepal: A 23-Year Retrospective Analysis of Bacteraemia
Source: Front Med (Lausanne). 2018 Sep 19;5:262. doi: 10.3389/fmed.2018.00262 (PMC6156253; doi:10.3389/fmed.2018.00262)
Supplement: Supplementary file 1 [file Presentation_1.pdf]

Table S1. Antimicrobials used in the susceptibility testing for this study

Antimicrobial	Abbreviation
Amikacin	AMK
Amoxicillin	AMX
Amoxicillin-clavulanic acid	AMC
Amoxycillin-sulbactam	AMX.SUL
Ampicillin	AMP
Ampicillin-sulbactam	SAM
Ampicillin-tazobactam	AMP.TZB
Azithromycin	AZM
Aztreonam	ATM
Cefepime-tazobactam	FEP.TZB
Cefixime	CFM
Cefoperazone	CFP
Cefoperazone-sulbactam	CFP.SUL
Cefotaxime	CTX
Cefoxitin	FOX
Cefpodoxime	CDP
Ceftazidime	CAZ
Ceftriaxone	CRO
Ceftriaxone-sulbactam	CRO.SUL
Cephalexin	LEX
Chloramphenicol	CHL
Ciprofloxacin	CIP
Clindamycin	CLI
Cloxacillin	CLO
Colistin	CST
Trimethoprim-sulphamethoxazole	SXT
Erythromycin	ERY
Gatifloxacin	GAT
Gentamicin	GEN
Imipenem	IPM
Linezolid	LZD
Kanamycin	KAN
Levofloxacin	LVX
Meropenem	MEM
Methicillin	MET
Nalidixic acid	NAL
Nitrofurantoin	NIT
Norfloxacin	NOR
Ofloxacin	OFX
Oxacillin	OXA
Penicillin	PEN
Piperacillin	PIP
Piperacillin-Tazobactam	TZP
Rifampicin	RIF
Teicoplanin	TEC
Tetracycline	TET
Tigecycline	TGC
Tobramycin	TOB
Vancomycin	VAN

Table S2. Antimicrobial categories used in this study

Name of categories	Members used in this study
Aminoglycosides	AMK, TOB, GEN, KAN
1st and 2nd generation cephalosporins	FOX, LEX
3rd and 4th generation cephalosporins	CFM, CFP, CTX, CPD, CAZ, CRO
3rd and 4th gen. ceph. + beta-lact. inhib.	FEP.TZB, CFP.SUL, CRO.SUL
Ansamycins	RIF
Carbapenems	IPM, MEM
Fluoroquinolones	CIP, GAT, LVX, NOR, OFX
Folate pathway inhibitors	SXT
Glycopeptides	TEC, VAN
Glycylcyclines	TGC
Lincosamides	CLI
Macrolides	AZM, ERY
Monobactams	ATM
Nitrofurans	NIT
Oxazolidinones	LZD
Penicillins	AMX, AMP, CLO, MET, OXA, PEN, PIP
Penicillins + beta-lactamase inhibitor	AMX.SUL, AMP.TZB, AMC, SAM, TZP
Phenicols	CHL
Polymyxins	CST
Quinolone	NAL
Tetracyclines	TET

Table S3. Intrinsic resistance to antimicrobials removed from MDR classification

Group/Organism	Non-susceptibility reported in our study but ignored for MDR profile [(a)]
Group-wide corrections for:	
Gram-positive	CST, NAL
Enterobacteriaceae	VAN, TEC, LZD, ERY, AZM, RIF
Additional organism-specific corrections for:	
Staphylococcus aureus	MET
Enterococcus spp.	CTX, AMK, TOB, GEN, KAN, SXT
Klebsiella spp.	AMP
Acinetobacter spp./*Pseudomonas spp.*	AMP, AMX, VAN, TEC, ERY, PEN
Citrobacter freundii	AMP
Proteus vulgaris	TET, AMP
Pseudomonas aeruginosa	CTX, CRO, TET, SXT, CHL

Table S4. Summary of bacteria positive blood cultures

Salmonella enterica	**n**	**% of *Salmonella enterica***	**% of all pos. cultures**
Salmonella Typhi	13,592	68.45	44.78
Salmonella Paratyphi A	6,057	30.50	19.96
Salmonella Paratyphi B/C/spp.	34	0.17	0.112
Salmonella Typhimurium/Enteritidis/spp.	174	0.88	0.573
Total	19,857	100	65.42
**Enterobacteriaceae non-*Salmonella***	**n**	**% of Enterob. non-*Salmonella***	**% of all pos. cultures**
Citrobacter diversus	3	0.07	0.01
Citrobacter freundii	28	0.69	0.09
Citrobacter spp.	74	1.84	0.24
Escherichia coli	1,228	30.48	4.05
Enterobacter aerogens	12	0.30	0.04
Enterobacter cloacae	7	0.17	0.02
Enterobacter spp.	1,672	41.50	5.51
Klebsiella oxytoca	19	0.47	0.06
Klebsiella pneumoniae	247	6.13	0.81
Klebsiella spp.	696	17.27	2.29
Proteus mirabilis	2	0.05	0.01
Proteus vulgaris	2	0.05	0.01
Proteus spp.	12	0.30	0.04
Serratia marcescens	1	0.02	0.00
Serratia spp.	26	0.65	0.09
Total	4,029	100	13.27

Table S4 (cont.)

**Gram-negative (other)**	**n**	**% of other Gram-negative**	**% of all pos. cultures**
Acinetobacter spp.	1,577	75.56	5.20
Aeromonas spp.	1	0.05	0.00
Alkaligens spp.	3	0.14	0.01
Campylobacter jejuni	2	0.10	0.01
Campylobacter spp.	2	0.10	0.01
Haemophilus influenzae	16	0.77	0.05
Haemophilus parainfluenzae	2	0.10	0.01
Haemophilus spp.	1	0.05	0.00
Moraxella catarhallis	1	0.05	0.00
Moraxella spp.	1	0.05	0.00
Neisseria gonorrhoeae	1	0.05	0.00
Neisseria meningitidis	39	1.87	0.13
Neisseria spp.	24	1.15	0.08
Providencia spp.	1	0.05	0.00
Pseudomonas aeruginosa	47	2.25	0.16
Pseudomonas spp.	257	12.31	0.85
Vibrio spp.	1	0.05	0.00
Unspecified Gram-negative rods	111	5.32	0.37
Total	2,087	100	6.88

**Gram-positive**	**n**	**% of Gram-positive**	**% of all pos. cultures**
Actinomyces spp.	8	0.18	0.03
Enterococcus faecalis	1	0.02	0.00
Enterococcus spp.	7	0.16	0.02
Coagulase negative *Staphylococcus*	1,465	33.45	4.83
Staphylococcus aureus	942	21.51	3.10
Staphylococcus epidermidis	1	0.02	0.00
Staphylococcus saprophyticus	2	0.05	0.01
Alpha haemolytic *Streptococcus*	340	7.76	1.12
Beta haemolytic *Streptococcus*	154	3.52	0.51
Non haemolytic *Streptococcus*	779	17.79	2.57
Streptococcus pneumoniae	627	14.32	2.07
Streptococcus pyogenes	8	0.18	0.03
Streptococcus viridans	14	0.32	0.05
Streptococcus spp.	3	0.07	0.01
Haemolytic *Streptococcus*	1	0.02	0.00
Gram-positive *rods*	28	0.64	0.09
Total	4,380	100	14.43

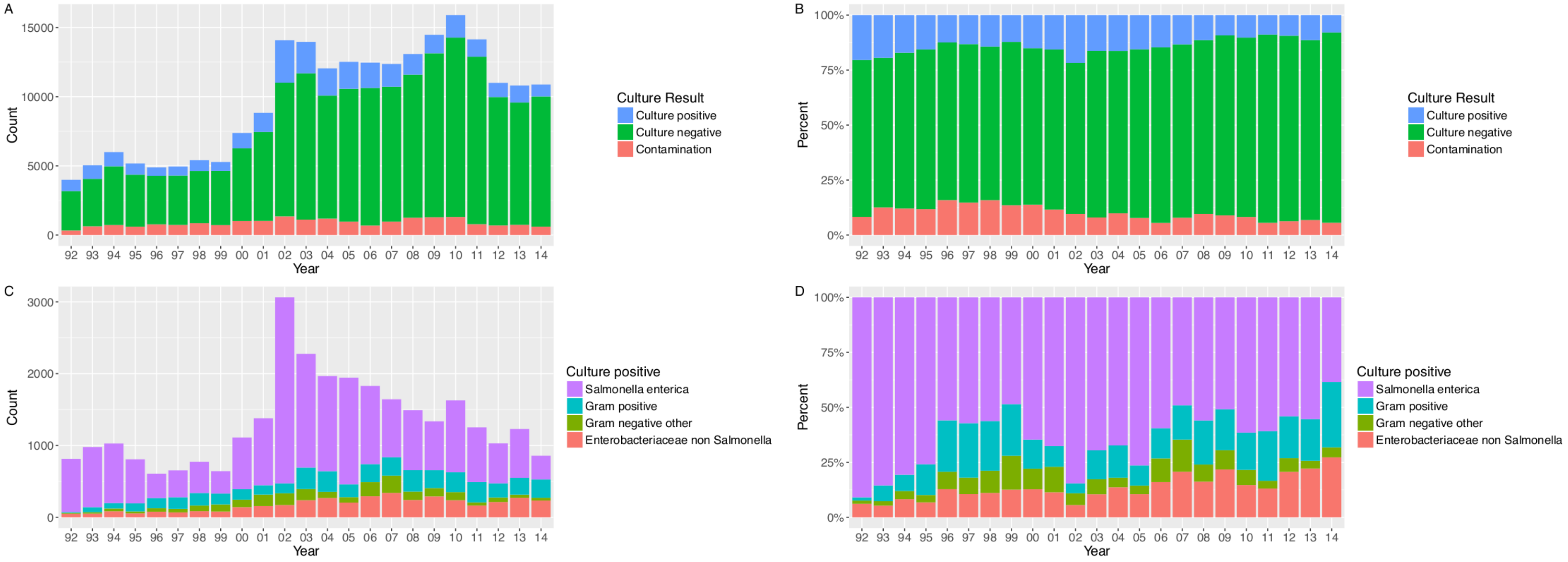

Figure S1. The annual distribution of positive blood cultures

The annual count (A) and distribution (B) of blood culture results from 1992 to 2014. The proportions of *Salmonella*, Enterobacteriaceae non-*Salmonella*, other Gram-negatives, and Gram-positive organisms isolated from positive blood cultures (C; count and D; distribution). Non-contaminated samples in which fungus was reported were excluded.

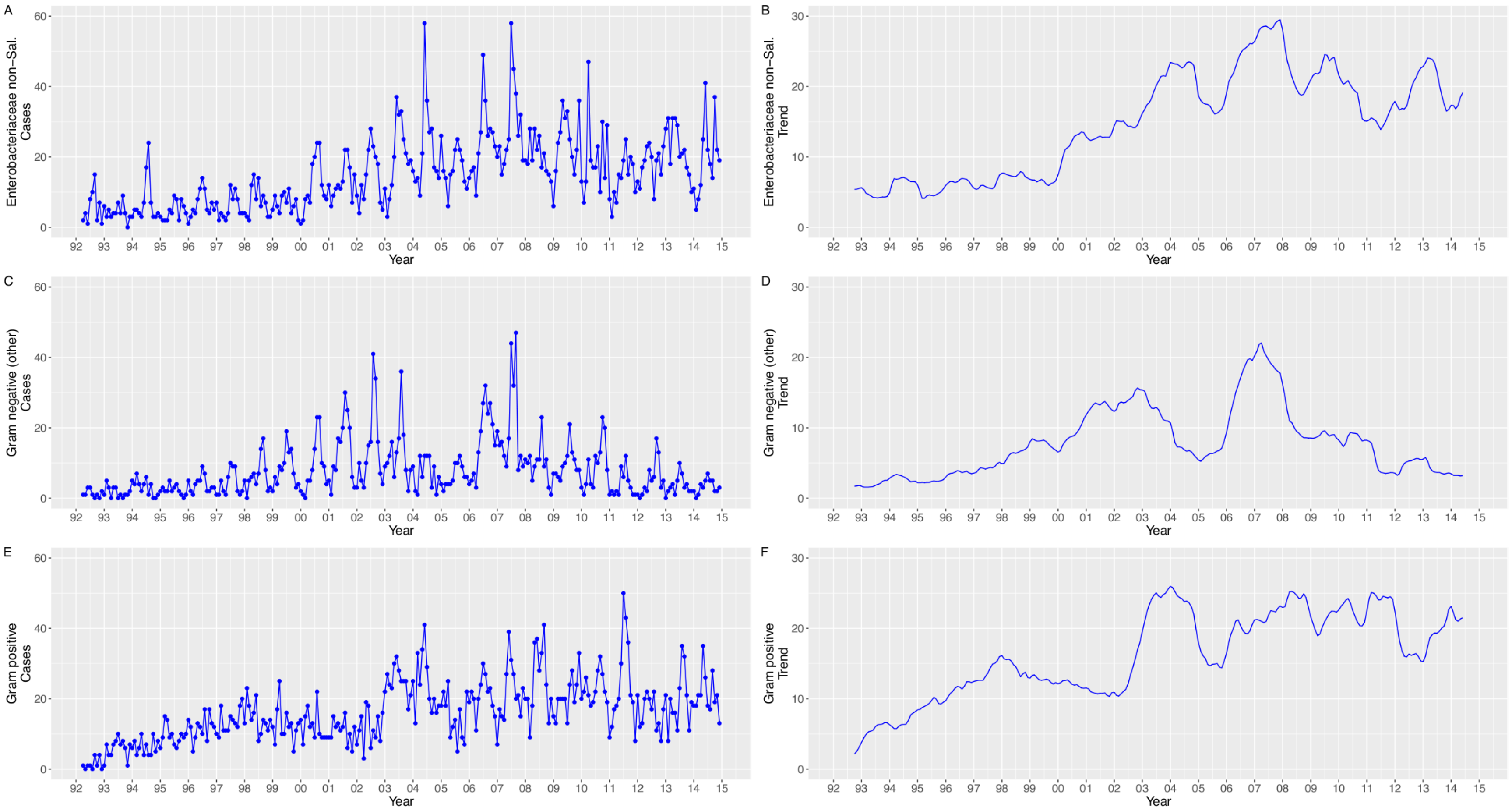

Figure S2. Time series for positive blood cultures

Monthly time series for Enterobacteriaceae non-*Salmonella* (A; number and B; time trends), other Gram-negatives (C; number and D; time trends), and Gram-positives (E; number and F; time trends) between April 1992 and December 2014.

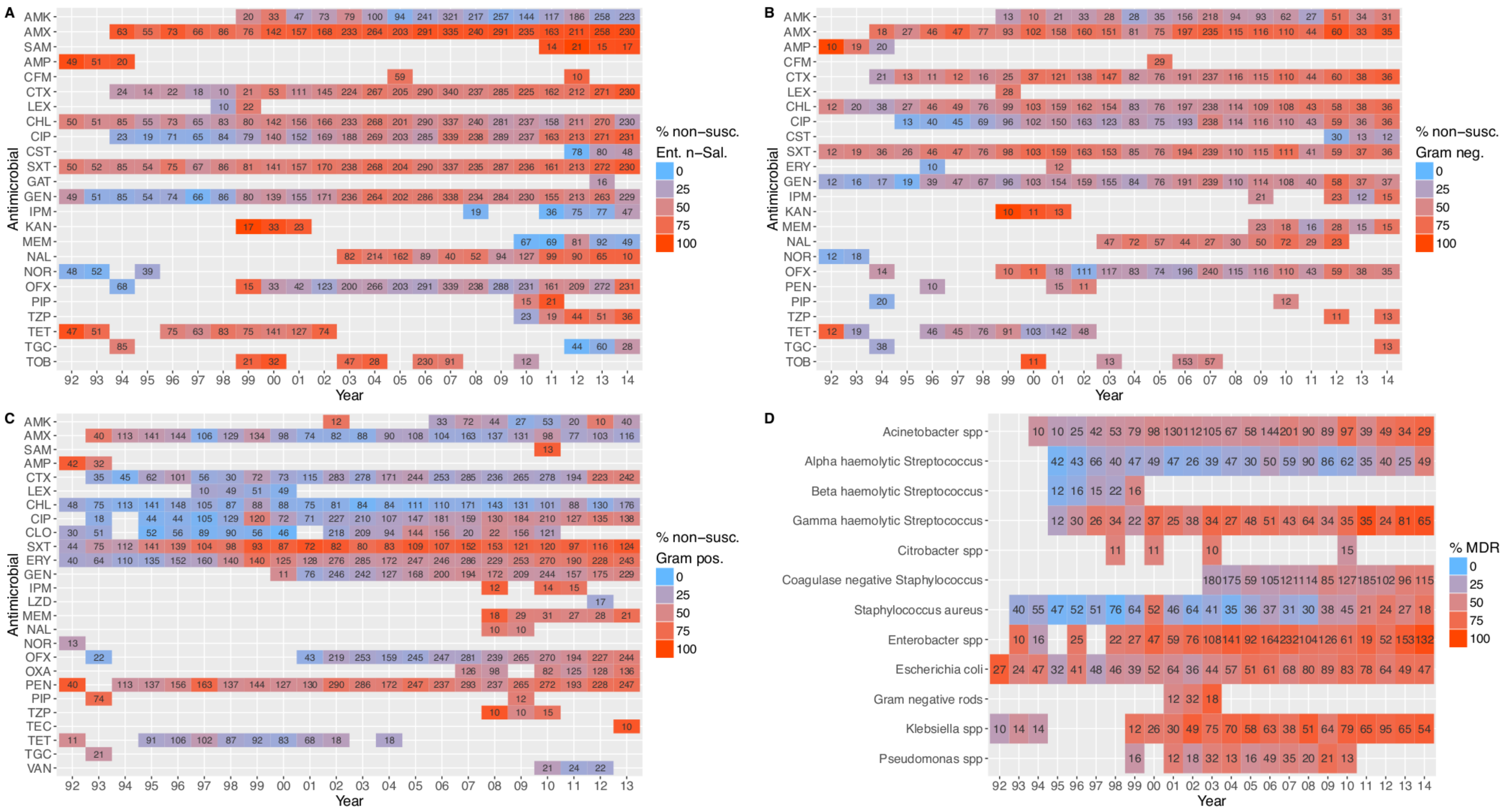

Figure S3. Autocorrelation for positive blood cultures

Autocorrelation function for monthly time series for Enterobacteriaceae non-*Salmonella* (A), other Gram-negatives (B), and Gram-positives (C); one lag represents a year divided in 12 months.

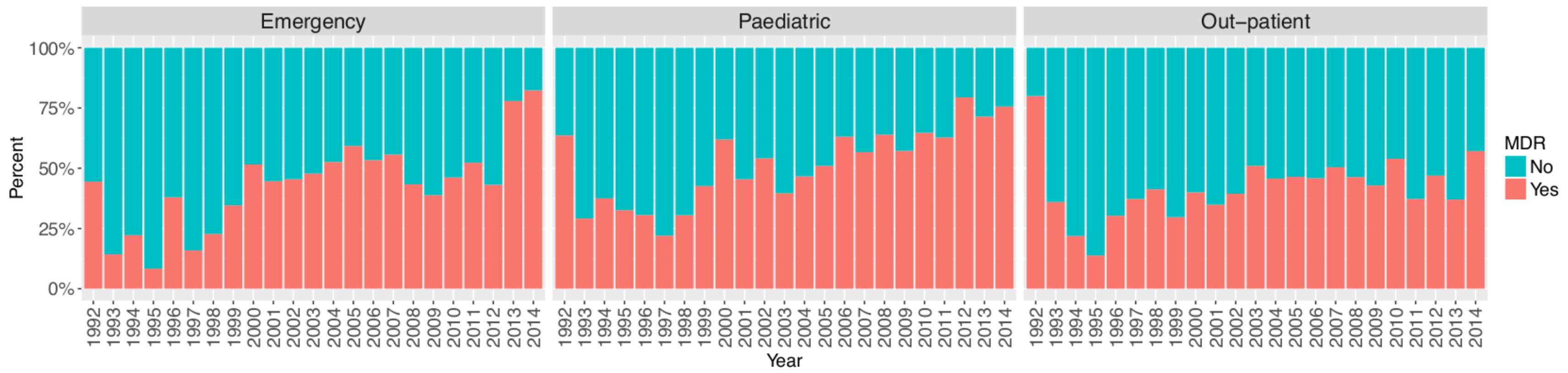

Figure S4. Multidrug resistance in positive blood culture from various hospital wards

Yearly counts of MDR and non-MDR organisms isolated from positive blood cultures, stratified by hospital ward in which the blood samples were collected between April 1992 and December 2014.
